# Leadership and Managerial Skills in Dentistry: Characteristics and Challenges Based on a Preliminary Case Study

**DOI:** 10.3390/dj10080146

**Published:** 2022-08-05

**Authors:** Maria Antoniadou

**Affiliations:** Dental School, National and Kapodistrian University of Athens, ACSTH by ICF and AC Accredited Coach, Thivon 2, Goudi, GR-11527 Athens, Greece; mantonia@dent.uoa.gr

**Keywords:** dental leadership, dentistry, dental management, emotional intelligence, dental leaders, soft skills, time management skills

## Abstract

Dentistry is changing rapidly in a dynamic and challenging mode and is incorporating digital technology, communication, and managerial skills for profitable enterprises. On the new dental horizon, the profession requires engaging and inspiring role models and leaders. Ιdentifying and developing human soft skills can improve quality issues and guarantee a sustainable dental business. The concept of leadership is a very complex and multidimensional phenomenon as observed from the current literature. In different commercial environments, there has been a huge discussion on the specific characteristics of an effective leader. In dentistry, the subject needs further investigation. This article aims to bring up the importance of dental leadership and highlights the need of identifying dental leaders committed to excellency. It also challenges the need for educational shift on dental leadership management. Finally, it aims to support and develop educational transformations based on positive preliminary data observed by incorporating a new, relevant subject in the dental curriculum.

## 1. Introduction

The word “leadership” is a derivative of the verb “lead”, which means “I am a leader and I lead others, who follow me willingly” [1]. Based on this etymology, leadership in the field of dentistry means the ability to influence or change the behaviors of one or more person. A leader is the one who provides the right environment for people to work. So, leadership is the process of influencing the thinking, feelings, attitudes, and behaviors of a small or large, formal or informal group of people in such a way that they voluntarily and willingly give their best [2]. There are multiple definitions of leadership (at least 350,000 within the academic literature) [3]. In dentistry, clinical leadership seems to be mentioned only once as “the skills required to provide effective patient care within a successful business” [4]. However, leadership has also other forms and seems to be more of a social process for goal attainment or an influence process [5]. Dental professionals are trained to integrate science in practice and education, have increased degrees of autonomy in judgments and clinical interventions, and are expected to be engaged in collaborative and interprofessional practices to achieve the best outcomes for patients, personnel, and organization [6]. Consequently, dental leaders are those who bring people together, help them communicate their needs and personal goals, inspire enthusiasm for a vision and a common mission, guide by example, take initiative, and ultimately gain the trust of the people around them [2]. They are also expected to substantially contribute to clinical outcomes through continuous quality improvement in patient care, create a supportive environment for their colleagues, and contribute to the development of their profession, healthcare systems, and healthcare policy [6,7,8].

Under this frame, the Association of Dental Education in Europe (ADEE) works on developing pan-European graduating clinicians who are trained in management and leadership [9]. Likewise, in Australia, leadership was embedded in the guide for professional competencies of newly qualified dentists [10] while the American Dental Association collaborated with experts in the field to provide a leadership educational framework [11]. A recent systematic review by Hanks et al. [5] and a doctoral dissertation thesis by Wardman [12] bring forward all leadership issues in the field of dentistry.

In this article, a discussion is evoked on the main leadership issues, the characteristics of an effective dental leader, and the approaches needed in dental education. Approaches are based on preliminary data retrieved from a new, relevant subject for leadership and managerial-skills enforcement in the dental curriculum of the Dental School of the National and Kapodistrian University of Athens, Greece.

## 2. Leadership Characteristics Needed in Dentistry

Characteristics needed for leadership evaluation could be addressed according to the organizational level of the dental business [5,13]. Starting from the strategic level, leadership is concerned about the systems in the (inter-) national level and regulating political issues concerning dentistry. In the second level, leadership is explored within dental associations, hospitals, or big dental units. Then, in the operational level, leadership is addressed within the team/group/unit emphasizing elements of practice (such as reception, surgery, aftersales service, etc.). In the tactical level that follows, leadership is referred to dyadic relationships mainly between colleagues. Finally, there is the individual level where leadership is needed for individual issues of one’s own evolution within the dental business framework.

The model of leadership that could be used in all the above levels defines more the competencies needed in the dental field. A simple, effective model is suggested to have five stages [14]. The first stage is that of position, where the leader has not yet attained real influence on people, and others follow him because of his role or position and because they are obliged to do so. No special competencies are needed in this stage since anyone can be the boss of a dental business. In the second stage, people agree to follow the boss because they want to. In this stage, good relationships with others are a crucial issue, where the leader should show not only his knowledge but how much he cares about others. So, in this stage, empathetic and emotional intelligence competencies are additionally needed. The third stage is that of production, where leaders help others to perform and bring results. In this stage, people follow the leader because of his results. Thus, he should have time management skills, creativity, problem solving and decision-making competencies. The fourth stage is the stage of evolution, where others follow the leader because of what he has done for them. Leaders in this stage are great not because of their power but because they concede power to others. They know how to create and communicate a vision, they are experienced, competent, supportive, respectful, self-determined, and consistent. The last seems to be a hidden stage in leadership evaluation in the healthcare business. Dentists, forced to attain productivity for themselves and their business, do not realize they should force the evolution of others in the unit/business. The last stage is where leaders gain respect of who they are, and others follow them for what they are or the values they represent.

Throughout the relevant literature, a specific set of skills or abilities are reported for all the above levels and stages of dental leadership [15,16,17,18,19,20,21,22]. A common key element of leadership in all the above levels is the value of accepting the self and others and be keen for excellency without following perfectionism, as mentioned in all theories about leadership (by Xenophon, Aristotle, Nietzsche, Blanchard, Covey, Collins, Goleman, and Barret, to name the most important ones who dealt with this issue). Leadership is also a practical matter. In other words, one simply needs to be able or willing to do himself what he asks others to do [23]. Many natural leaders have been already reported. However, although there are certain genetic qualities that can help accelerate the development of this ability, there is insufficient evidence that leadership exists in the human DNA [24]. In defense of this, it can be noted that gender does not play a role in the dynamics of leadership [25,26]. It also seems there are no clear features that predetermine the prospect of leadership, but people who are adaptable, social, and ambitious have a good chance of becoming leaders. It also appears that IQ index contributes less than 5% to the possibility of becoming a leader [24]. So, it is rather generally accepted that leadership behaviors stem from a combination of a small percentage of genetic and a large percentage of environmental factors, as is the case with other abilities and practices. It is worth noting that while some people may be born with these dynamics, they only develop their leadership skills by 30–60% over time [27].

## 3. Theories about Leadership and Suggested Competencies

The theories about leadership are many and through them one can outline the necessary characteristics that must distinguish leaders.

### 3.1. Theories of Individual Characteristics

These theories are based on the notion that the characteristics of a leader are related to his effectiveness. In 1981, Stogdill cited as essential characteristics, boldness, self-confidence, readiness, the ability to influence the behavior of other people, responsibility, and acceptance [28]. It was later reported that some positive elements of the personality of people with managerial positions such as optimism, determination, and self-discipline were directly related to the degree of success in their work [27]. The personality model of five factors, namely, extroversion, conscientiousness, affability, receptivity to experience, and lack of neuroticism, has been also reported. Research data show that extroversion has a particularly positive effect on leadership issues [29].

### 3.2. Behavioral Theories

Leadership ability, according to behavioral theories, is not a hereditary and innate process, but a complex, acquired skill that can be developed in different environments in many ways and depending on circumstances. Thus, there are three main styles of leadership behavior: (a) the authoritarian (the leader makes the decisions himself, without the participation of the group, which has only an executive role), (b) the democratic (the leader cooperates with the group and accepts advice and participation of others in decision making), and (c) the empowering leader (limited role and group decisions) [30]. Later, the behavior of a leader was given emphasis rather than his traits [2,31,32]. The managemental grid model that followed [33] mentioned two leadership dimensions: X, the interest in people, and Y, the interest in service. For the professional dentist, each dimension is graded from one to nine and the combination resulting from the grades of the two dimensions leads to a certain style of leadership. The larger the area determined by the two dimensions, the greater his leadership skill [2].

### 3.3. Leadership Theories Related to the Situation

According to theories related to specific situations, there are two leadership dimensions: (a) toward people and (b) toward tasks [34]. Every situation needs a combination of styles depending on the specific reality one must face [2]. Task-oriented leadership approaches are generally most effective in health enterprises where: (1) the team is limited by resources or time, (2) there is disruption in the structure and an order needs to be restored, (3) the leader works with many or nonqualified team members. People-oriented leadership approaches are more effective in the administrative tasks of the business. So, they are needed when there is an opportunity to develop the leadership skills of the staff and/or the need to motivate through new experiences and greater responsibility. We can also work on a more people-centered basis when working with a highly experienced, skilled, and competent team [35]. The level of maturity of the followers basically defines the most appropriate leadership style [36] as well as their degree of satisfaction of expectations from the leader [37,38]. It was finally reported that leaders can be effective in one situation but ineffective in another [39]. As it seems, the conditions prevailing in the environment act as a catalyst in the leadership style that one adopts [40].

### 3.4. New Approaches to Leadership

Modern approaches suggest the motivation of people derives from sharing a common vision and the sense of belonging to a common team. Recognizing the needs of partners and cultivating emotional intelligence are also important factors [41,42,43]. Under this scope, management of a dental business seems a different concept than leadership. Management is just doing things right, leadership is doing the right thing [44]; and management is transactional, while leadership is transformational [45,46,47]. In the field of health entrepreneurship, the approach to superleadership is also special [48]. Based on this theory, leaders should create a pyramid of leadership through the promotion of new leaders. By this sense, dental leadership principles reinforce dental management and make it more productive and effective. 

## 4. Leadership Models in Healthcare

A well-used leadership model in the health sector is the nine-dimensional model of Britain’s National Health Service (NHS) [49] based not on the behavior of the followers but on a combination of emotional expressiveness, self-confidence, self-determination, resilience, and freedom from internal conflicts of the leader [35,38,43]. Setting the tone and culture of an organization is intertwined with resolving conflict, giving feedback, and creating a nonjudgmental learning environment for all [45,50], where there is no intimidation or fear [51]. A total of 30 leadership core competencies within (seven) leadership domains are discussed in Table 1, endorsed by the dental field [6,51,52,53,54,55,56,57,58,59,60].

## 5. Preliminary Report on a Leadership Educational Approach in Dental Education

In the dental school of the National and Kapodistrian University of Athens, Greece, a new course was introduced on leadership and dental management enhancement, pilot-tested to dental students in their eighth semester of undergraduate studies. The course was organized for two consequent years, since 2021, and was held during the spring semester. The course “Application of humanities and basic principles of coaching in dentistry” addresses leadership issues, humanities (philosophy, positive psychology), communication and time management skills, managerial and personal branding, and marketing issues. The educational process consisted of 13 weekly seminars of two hours each. The first hour explored theoretical issues of the modules, while the second hour was used for practical and experiential exercises derived from the main theme of the module. The 13 modules of the course specifically addressed the following issues: (1) vision, values, and goal setting in dental management, (2) time management in dentistry, (3) philosophical approach and ethics in dentistry, (4) the Aristotelian values in dental ethical leadership, (5) positive psychology introduction for health care professionals, (6) emotional intelligence reinforcement in dentistry, (7) motivation and mechanism of behavior changes in healthcare, (8) meditation in healthcare for resilience enhancement, (9) effective communication in dentistry, (10) setting healthy limits in dental business, (11) management of the success and failure in dental business, (12) management of fear in the dental business, and (13) strategic thinking for decision making, dental management and marketing in dental business.

Students were enrolled voluntarily in the course between March 2021 and June 2022 (N1 = 22). In the beginning of the course (beginning of the eighth semester each year–1st March) (t_0_), students were asked on a voluntary basis to fill a questionnaire based on (a) time management, [61], (b) the level of emotional intelligence and resilience (FEEL) [2], and (c) collaboration issues [62,63]. The same questionnaire had to be filled again at the end of the course (end of the eighth semester, middle June each year) (t_1_). Each of the students had chosen a personal code to anonymously fill the questionnaire in both time periods.

A total of 14 questionnaires were initially collected at t_0_ for both academic periods (initial response rate 63.6%). A total of 12 questionnaires were finally collected at t_1_ from the same participant (final response rate 54.5%). The most interesting preliminary findings concerned 80% improvement in time-management skills, especially procrastination and prioritization. A 72% improvement was observed in enhancing collaboration issues, a 68% improvement on addressing values and setting goals, and a 66% improvement on decision making. A 58% improvement was finally reported on emotional intelligence skills, self-confidence, and self-respect issues. Even though seminars and exercises were not organized in a 1:1 coaching session base but in a group mode, all participants reported positively on the importance of the course and the benefits gained over many leadership and management issues. Some limitations of this approach were the semester that the subject was launched (eighth) when all students were trying to cope with newly addressed clinical skills, had no breaks between courses, and were reluctant to choose another course with no obvious result at this period of their studies. Additionally, in the first year of running the course, seminars were introduced via e-learning due to the COVID-19 pandemic, possibly diminishing the impact of personal involvement in the issues discussed and the exercises performed within the group.

## 6. Discussion

Sustainability issues of the dental profession cause the need to treat every single graduating and graduated dentist as a potential leader toward himself, patients, and colleagues. Unfortunately, so far, technical skills development is focused on, while leadership training remains behind. Dental schools and curricula represent the best opportunity to start training the future generation of dentists who will be able to perform in an excellent clinical and professional manner.

Leadership educational interventions are suggested to include a scaled module [64,65]. Initially, dental students could be introduced to basic theoretical issues of emotional intelligence and time management while, in the meantime, they perform different educational activities in small working groups. More specifically, the educational framework should encourage dental students’ engagement in (a) teamwork activities where all members should have specific roles and responsibilities, (b) working groups for active listening and emotional intelligence improvement, (c) mentoring by trained academic staff, and (d) coaching by trained coaches to surpass negative personal beliefs, deal with stress issues, or manage failure. Under this scope, the role of an educator, coach, or mentor should be enhanced from the beginning of studying dentistry until graduation. The mentor could present students with motivation to achieve not only clinical excellency but also strategic leadership excellency in all fields of human resources management addressed in dentistry [66,67]. The desire to stand out, to become better, and to make a difference in the dental field is inherent. It leads down the path of personal development, making students better dentists and entrepreneurs [68,69,70,71].

By this approach, the accountable future dental professional leader will ensure excellence and quality assurance in the dental units by addressing: (1) people and managing talent [10], (2) vision and legacy of success in the system [72], (3) culture of excellent clinical performance and ethics [73], (4) quality assurance policies [74], (5) effective teamwork [13,75], (6) quality of patient care [5], (7) inspiring relationships based on mutual trust [2,76], (8) quality and ensuring healthy dental workplaces [51], (9) well-being at work [2,51,70,77,78], and (10) reduction of bullying in the dental work field [19,77,78].

Although limited, data from the above educational experience are optimistic on the changes expected in students’ mindsets on leadership and dental management issues. Information retrieved in a more extended scale from running the course will be addressed accordingly in the future. No doubt this is an interesting and promising research field and will excel human resources management education in dentistry. Future research should be encouraged to propose and define specific techniques and modules concerning human soft skills enhancement for next generation dental professionals.

## 7. Conclusions

Dental leadership can be learned and exercised in modern educational programs. Further research is needed to provide data on leadership teaching approaches based on scaled modules and measuring the core leadership competencies before and after interventions.

## Figures and Tables

**Table 1:** Domains, competencies, and characteristics of leadership.

Domains	Competencies	Characteristics, Actions, Results
**Clinical Leadership domain**	Provides leadership to the healthcare team to promote health, facilitate self-care management, optimize patient engagement, and prevent future oral health problems	Acting as a resource person, preceptor, mentor/coach, and role model demonstrating critical and reflective thinking
	Assumes, as a clinical expert, a leadership role in establishing and monitoring standards of practice to improve client care	Establishing intra- and interdisciplinary peer supervision and review, taking decisions, and commitment
	Analyzes organizational systems for barriers and promotes enhancements that affect clients’ oral healthcare status	Strategic and analytical thinking, taking decisions, and concept mapping
	Engages in advanced dental practice and provides leadership for evidence-based practice	Identifying current relevant scientific information, translation of research in practice, forming the evaluation of practice, improving the reliability of healthcare practice and outcomes, participating in collaborative research, and endorsement of continuing education
	Provides leadership and acts as a liaison with other health agencies and professionals	Participating in assessing and evaluating healthcare services to optimize outcomes for patients/clients/communities and taking initiatives
	Collaborates with healthcare professionals, including physicians, and others to plan, implement, and evaluate an improvement opportunity	Skills of communication and collaboration, selecting the right team members, transactional analysis, and taking initiatives
	Aligns practice with overall organizational/contextual goals	Strategic thinking and organizational/managerial skills
	Guides, initiates, and provides leadership in (1) the development and implementation of standards, practice guidelines, and quality assurance, (2) education, and (3) research initiatives	Open-minded philosophy, excellency endorsement, and analytical thinking
**Professional Leadership domain**	Participates in professional organizations and activities that influence dental practice	Socially friendly personality, time-management skills, and knowledge of prioritization of activities
	Provides leadership in the development and integration of the dental practitioner role within the healthcare system	Taking initiatives, caring for social presence, and voluntarism
	Assumes responsibility for his or her own professional development by pursuing education, participating in professional committees and work groups, and contributing to a work environment where continual improvements in practice are pursued	Excellency endorsement and consistency
	Employs consultative and leadership skills with intraprofessional and interprofessional teams to create change in oral health care	Participation in mastermind’s groups, innovation endorsement, and reflective thinking
	Participates in peer-review activities, e.g., publications, research, and practice	Studying skills and good knowledge of foreign languages
	Participates in relevant networks; regional, national, and international	Collaboration skills, philosophy of sharing, and contribution
**Health Systems Leadership domain**	Contributes to development, implementation, and monitoring of organizational performance standards	Review of current needs of the market, economic analysis performance, and vision setting
	Assumes a leadership role of an interprofessional healthcare team with a focus on the delivery of patient-centered care and the evaluation of quality and cost-effectiveness	Managerial skills, quality assurance policy, endorsement of excellency, and commitment to working collaboratively
	Demonstrates a leadership role in enhancing group dynamics and managing group conflicts within the dental business	High emotional intelligence performance, communication skills, and defining personal and professional limits
	Plans and implements training and provides technical assistance to staff members and personnel within the business and in other community and governmental agencies and organizations	Educator skills and willingness to share knowledge
	Creates a culture of ethical standards within organizations and communities	Holds values and ethics; existence of a personal ethic guide
	Identifies internal and external issues that may impact delivery of essential dental health services	Knowledge of the market, prediction of the flow, and quantity and quality of events that matter
**Health Policy Leadership domain**	Guides, initiates, and provides leadership in policy-related activities to influence practice, health services, and public policy	Legal and ethical code knowledge in the field of dentistry, decisiveness, and consistency
	Articulates the value of dentistry to key stakeholders and policymakers	Legal and ethical code knowledge in the field of dentistry
**Clinical and Dental Health Systems Leadership domain**	Uses advanced communication skills/processes to lead quality improvement and patient safety initiatives	Positive communication skills and effective listening
	Employs principles of business, finance, economics, and health policy to develop and implement effective plans for practice-level and/or systemwide practice initiatives that will improve the quality-of-care delivery.	Managerial and economic analysis skills
	Advocates for and participates in creating an organizational environment that supports safe client care, collaborative practice, and professional growth	Empathetic behavior, excellency endorsement, and collaborative attitude
	Creates positive, healthy (work) environments and maintains a climate in which team members feel heard and safe	Justice and reward endorsement, give equal opportunities to all team members, and inspiring motivation
**Professional and Dental Health Systems Leadership domain**	Prepares, through mentoring and coaching, future generations of dentists	Coaching and empathetic skills, intention of collaboration and group enforcement, and positive feelings for group excellency
**Clinical, Dental Health Systems, and Health Policy Leadership domain**	Provides leadership in the evaluation and resolution of ethical and legal issues within dental systems relating to the use of information, information technology, communication networks, and patient care technology	Technical skills in technology and sufficient knowledge and use of social media

## Data Availability

Data retrieved upon request.
